# Acknowledgement of manuscript reviewers 2015

**DOI:** 10.1186/s40945-016-0018-0

**Published:** 2016-06-08

**Authors:** Marco Baccini

**Affiliations:** Azienda Sanitaria di Firenze and Florence University, Firenze, Italy

## Abstract

The editors of *Archives of Physiotherapy* would like to thank all our reviewers who have contributed to the journal in Volume 5 (2015).

**Margit Alt Murphy**

Sweden

**Marco Barbero**

Switzerland

**Christoph Michael Bauer**

Switzerland

**Lucia Bertozzi**

Italy

**Gennaro Boccia**

Italy

**Shaun Boe**

Canada

**Arianna Bortolami**

Italy

**Jacopo Buzzi**

Italy

**Alicia Calle**

Spain

**Davide Cattaneo**

Italy

**Erik Cattrysse**

Belgium

**Alessandro Chiarotto**

The Netherlands

**Anna Christakou**

Greece

**Chad Cook**

US

**Davide Corbetta**

Italy

**Stefano Corna**

Italy

**Rogelio Coronado**

US

**Sandro Cortini**

Italy

**Adrienne Davidson**

Italy

**Mauro Di Bari**

Italy

**Mark Elkins**

Australia

**Heidi Engel**

US

**Brigitte Erdmann**

Switzerland

**Cathy Evans**

Canada

**Deborah Falla**

Germany

**Francesco Ferrarello**

Italy

**Silvano Ferrari**

Italy

**Sandro Fioretti**

Italy

**Ferrarello Francesco**

Italy

**Jose Frantz**

South Africa

**Simon French**

Canada

**Alessio Gallina**

US

**Tommaso Geri**

Italy

**Andrew Guccione**

US

**Jean Hay-Smith**

New Zealand

**Susan Hillier**

US

**Anne-Marie Hutchison**

UK

**Rebecca Kerney**

UK

**Niina Kolehmainen**

UK

**Marta Lazzeri**

Italy

**Antonio La Torre**

Italy

**Diego Leoni**

Switzerland

**Nicola Maffiuletti**

Switzerland

**Alessandro Mannoni**

Italy

**Inzitari Marco**

Spain

**Elisa Martín-Arévalo**

Spain

**Michael Mccarthy**

US

**Paul Mclaughlin**

UK

**Rita Neviani**

Italy

**Peter Nydahl**

Germany

**Nina Rydland Olsen**

Norway

**Matteo Paci**

Italy

**Selina Parry**

Australia

**Elisa Pelosin**

Italy

**Daniele Piscitelli**

Italy

**Giuseppe Plebani**

Italy

**Giacomo Rossettini**

Italy

**Claudia Sarno**

Italy

**Micaela Schmid**

Italy

**Jochen Schomacher**

Switzerland

**David Simoni**

Italy

**Leon Straker**

Australia

**Anne Swisher**

US

**Andrea Tettamanti**

Italy

**Holm Thieme**

Germany

**Louis Tremblay**

Canada

**Andrea Turolla**

Italy

**Andrea Ungar**

Italy

**Stefano Vercelli**

Italy

**Markus Wirz**

Switzerland

**Daniela Zani**

Italy

